# Hyperbilirubinemia following lenalidomide administration

**DOI:** 10.1002/ccr3.1471

**Published:** 2018-03-13

**Authors:** Veronica Azmy, Natalia Neparidze

**Affiliations:** ^1^ Yale University School of Medicine New Haven CT 06510; ^2^ Yale University School of Medicine Smilow Cancer Hospital, New Haven CT 06510

**Keywords:** chemotherapy, Gilbert's syndrome, hyperbilirubinemia, lenalidomide, multiple myeloma

## Abstract

Asymptomatic hyperbilirubinemia in a patient with no underlying liver disease or renal impairment while on lenalidomide therapy may be attributable to the unmasking of previously undiagnosed Gilbert's syndrome, as previously shown in the literature. The hyperbilirubinemia should resolve after discontinuation of the drug.

## Introduction

This is the case of a 72‐year‐old man with asymptomatic IgG lambda multiple myeloma who developed isolated elevation in indirect bilirubin after one cycle of lenalidomide therapy. With no evidence of hemolysis, liver disease, or renal impairment, it appeared that lenalidomide had unmasked Gilbert's syndrome.

We report the case of a 72‐year‐old Caucasian man with asymptomatic IgG lambda multiple myeloma (MM) who was being treated on a randomized phase III clinical trial of lenalidomide versus observation for asymptomatic MM.

Lenalidomide is an immunomodulatory agent with anti‐angiogenic and antineoplastic properties that inhibits proliferation and induces apoptosis of certain hematopoietic tumor cells including those of MM, mantle cell lymphoma, and myelodysplastic syndromes 1, 2. Its mechanism of action is multifaceted and includes activation of T cells and NK cells, increasing the number of NK cells, and inhibiting pro‐inflammatory cytokines such as TNF‐*α* and IL‐6. In multiple myeloma cells, the combination of lenalidomide and dexamethasome works synergistically to inhibit cell proliferation and induce apoptosis.

Our patient received Cycle 1 of lenalidomide 25 mg orally daily for 21 days. After 3 weeks of therapy, he presented prior to Cycle 2, Day 1 of lenalidomide with hyperbilirubinemia. Laboratory studies showed a total blood bilirubin of 3.88 mg/dL, consistent with grade 3 hyperbilirubinemia.

At this point, our greatest concern was the possibility of lenalidomide‐induced hepatotoxicity. Approximately 2% of patients with MM developed hepatotoxicity in clinical trials with lenalidomide 3. It has been previously described in the literature in several cases 4, and its occurrence has mainly been reported in those with renal insufficiency or with underlying liver disease.

The patient did not have a history of known liver disease, and he remained asymptomatic. His renal function also remained stable within the normal range. All other liver function tests, including transaminases, were within normal limits. The hyperbilirubinemia was largely due to indirect bilirubin elevation, as direct bilirubin was normal.

Given that his hyperbilirubinemia was indirect in nature, hemolysis was our next concern. Thrombotic microangiopathy (TMA), which some term cancer‐related microangiopathic hemolytic anemia (CR‐MAHA) 5, can be associated with multiple myeloma but is exceedingly rare, presents with thrombocytopenia, and is usually related to treatment with bortezomib 6. Our patient did not have thrombocytopenia, so this was lower on our differential. In addition, we could not rule out the possibility of underlying liver disease as this was his first presentation with hyperbilirubinemia and he did not have any previous imaging.

Lenalidomide treatment was held. Indirect and total bilirubin levels (3.68 mg/dL) were repeatedly elevated during the following week. Direct bilirubin, AST, ALT, alkaline phosphatase, and LDH remained within normal limits. There was no evidence of hemolysis, as hemoglobin and LDH were normal and direct Coombs test was negative. Ultrasound of the liver demonstrated normal echotexture, no biliary ductal dilation, or focal masses.

Based on the laboratories and imaging studies, the patient did not have hemolytic anemia or underlying liver disease. We had to broaden our differential to other etiologies of asymptomatic hyperbilirubinemia, such as Gilbert's syndrome (GS). Gilbert's syndrome occurs in 3–10% of the general population and is characterized by mild indirect nonhemolytic hyperbilirubinemia in the absence of liver disease. It is caused by a variant in UGT1A1, a dinucleotide repeat polymorphism in the promoter region of the gene that lowers gene expression and reduces the enzyme's activity. An individual can be a homozygote or a heterozygote for the variant, with homozygotes having a higher risk of hyperbilirubinemia due to decreased enzyme activity. The manifestation of GS requires both the genetic variant and a precipitating factor, such as medication administration, illness, or fasting. In a case report by Simondsen and Kolesar, their patient, a heterozygote for UGT1A1, exhibited asymptomatic elevation in unconjugated bilirubin in response to lenalidomide 10 mg orally daily for a 21‐day cycle 7. As the patient remained asymptomatic and was demonstrating only an isolated indirect hyperbilirubinemia, we considered the possibility that lenalidomide had unmasked underlying GS.

We suspected that lenalidomide had unmasked Gilbert's syndrome in this individual, as previously described in the literature. Cycle 2, Day 1 of treatment was delayed by 1 week, at which time the hyperbilirubinemia resolved. He resumed Cycle 2 of lenalidomide at a reduced dose of 15 mg daily. During the subsequent treatment course, the patient exhibited only grade 1 hyperbilirubinemia and was able to continue with treatment. For this reason, we concluded that the unmasking of GS by lenalidomide was dose‐dependent.

## Commentary

Lenalidomide, a thalidomide analog, is an immunomodulatory agent with anti‐angiogenic and antineoplastic properties used for the treatment of multiple myeloma. We presented the case of a 72‐year‐old man with asymptomatic IgG lambda multiple myeloma who developed isolated elevation in indirect bilirubin after one cycle of lenalidomide therapy, and which resolved after discontinuation of the drug. Given that the patient was asymptomatic, had no transaminitis, no evidence of underlying parenchymal liver disease, and no evidence of hemolysis, it appeared that lenalidomide had unmasked underlying Gilbert's syndrome, as previously described in the literature.

There has been one report of lenalidomide unmasking GS in an individual who was heterozygote for the TA7 allele, which can decrease expression of uridine diphosphate glycuronosyl transferase 1A1, the enzyme responsible for conjugation of bilirubin 8. The mutation, A (TA) 7TAA, is thought to be the main cause of the syndrome in Caucasians; however, more than 100 different mutations have been implicated in GS, including enhancer polymorphisms that lower transcriptional activity, as well as missense mutations which affect enzyme function 9.

The patient described developed grade 3 indirect hyperbilirubinemia while remaining asymptomatic and without other evidence of hepatotoxicity. His presentation is inconsistent with previously reported lenalidomide‐induced hepatotoxicity, which has been reported in patients with renal insufficiency. Our patient had normal renal function throughout his treatment course. His testing for the UGT1A1 gene variant was negative; however, he may harbor another mutation described in the literature. The patient subsequently went on to receive lenalidomide at a lower dose with waxing and waning minor elevations in total bilirubin, indicating that perhaps the risk of hyperbilirubinemia is dose‐dependent. Further research looking into the mechanism by which lenalidomide induces hyperbilirubinemia is warranted.

## Authorship

VA: compiled and reviewed the literature, wrote and edited the manuscript, and acted as the corresponding author. NN: guided and compiled the literature review, offered clinical experience, and wrote and reviewed the manuscript.
